# Endometriosis - on the intersection of modern environmental pollutants and ancient genetic regulatory variants

**DOI:** 10.1038/s41431-025-01977-9

**Published:** 2025-11-20

**Authors:** Amelia Warren, Demetra Andreou, Dean Warren, Jack Wieland, Anna Mantzouratou

**Affiliations:** 1 Department of Life and environmental Sciences, Bournemouth University, Bournemouth, BH12 5BB England; 2 Independent Researcher, Poole, UK

**Keywords:** Gene expression, Evolutionary biology, Endocrine reproductive disorders

## Abstract

Endometriosis is a chronic, estrogen-driven inflammatory disorder affecting approximately 10% of reproductive-aged women globally. Despite increasing genomic insights into advanced-stage disease, the genetic underpinnings of early-stage endometriosis remain poorly understood, limiting opportunities for timely diagnosis and intervention. This study explores the contribution of regulatory variants, including those derived from ancient hominin introgression, and their interaction with modern environmental exposures in shaping endometriosis susceptibility. We conducted a dual-phase literature review to identify genes implicated in endometriosis pathophysiology and endocrine-disrupting chemical (EDC) sensitivity. Five genes (*IL-6, CNR1, IDO1, TACR3*, and *KISS1R*) were selected based on tissue expression, pathway involvement, and EDC reactivity. Whole-genome sequencing (WGS) data from the Genomics England 100,000 Genomes Project were analysed in nineteen females with clinically confirmed endometriosis. Variant enrichment, co-localisation, and linkage disequilibrium analyses were conducted, and functional impact was evaluated using public regulatory databases. Six regulatory variants were significantly enriched in the endometriosis cohort compared to matched controls and the general Genomics England population. Notably, co-localised *IL-6* variants rs2069840 and rs34880821—located at a Neandertal-derived methylation site—demonstrated strong linkage disequilibrium and potential immune dysregulation. Variants in *CNR1* and *IDO1*, some of Denisovan origin, also showed significant associations. Several of these variants overlapped EDC-responsive regulatory regions, suggesting gene-environment interactions may exacerbate risk. These findings propose a novel perspective of endometriosis susceptibility, in which ancient regulatory variants and contemporary environmental exposures converge to modulate immune and inflammatory responses. This integrative approach identified new potential biomarkers for early-stage detection of endometriosis.

## Introduction

Globally, ten percent of reproductive-aged women have endometriosis, a heterogeneous gynaecological disease driven by estrogen signalling [1]. Endometriosis can be difficult to diagnose due to limited diagnostic tools, contributing to misdiagnosis and delays. Diagnosis can take up to eleven years between symptom onset and diagnosis [2]. Fifty percent of diagnosed women medically reported severe pelvic pain during adolescence that went untreated [3].

Endometriosis is potentially a multifactorial disease and may involve a complex system of immunological, environmental, hormonal, and genetic factors. Studies have suggested a dampened immune response in endometriosis patients due to estrogen dominance, triggering pro-inflammatory factors and altering immune cell functions. This fuels chronic inflammation and prevents cell death, promoting endometrial lesion growth [4]. Furthermore, a study using twins found a heritability component, with genome-wide association studies (GWAS) suggesting a genetic (47%) and environmental (53%) contribution to endometriosis predisposition [5]. Environmental predisposition from modern industrial pollutants and chemicals such as endocrine-disrupting chemicals (EDCs) may play a role in endometriosis development. EDCs imitate hormones and block naturally occurring hormones from binding to receptors. This can interfere with physiological processes, including the reproductive system [6]. Current GWAS have collectively identified forty-two single nucleotide polymorphisms (SNPs) linked to endometriosis, some of which are associated with pain perception and maintenance and advanced endometriosis [7–9], however, none of these SNPs predict early endometriosis stages, hindering increased risk assessment accuracy and early diagnosis preventing complications like infertility.

Despite advancements in identifying endometriosis genes, research largely focuses on advanced stages and comorbidities, rather than disease onset, leading to a diagnosis of earlier stages and prevention of endometriosis to remain elusive. Understanding genetic risk and gene-environment interaction in early endometriosis is key to improving endometriosis management and preventing complications like infertility and gynaecologic cancers. This study aims to bridge the gap between genetics and environmental risk factors, providing a more comprehensive perspective of endometriosis susceptibility and identifying potential biomarkers for early-stage detection.

## Materials and methods

### Literature searches

A two-phase systematic literature review was conducted using PubMed and Web of Science to identify genes and genomic markers implicated in endometriosis pathophysiology and their interaction with environmental exposures, particularly EDCs.

#### Literature selection criteria

Inclusion Criteria: original studies focusing on genomic/genetic analysis, or genome-wide association design, only human participants (no other species), patients with a diagnosis of endometriosis for at least a year, patients aged between eighteen and forty-three years at the time of recruitment.

Exclusion Criteria: review studies, studies including participants with other types of female infertility, participants without an endometriosis diagnosis, participants with additional illnesses and diseases which could affect result outcomes.

A literature search investigating environmental risk factors for endometriosis and a review of their impact on signalling pathways and related genes was conducted. The key words used were; “endometriosis” and “exposure to endocrine disrupting chemicals”, “endocrine disrupting chemicals”, “exposure to pesticides”, “pesticides”, “personal care products”, “cosmetics”, “exposure to heavy metals”, “heavy metals”, “exposure to radiation”, “radiation”, “exposure to toxins”, “toxins”, “chemicals”, “plastics”, “exposure to pollution”, “pollution”, “exposure to air pollution”, “air pollution”, “exposure to water pollution”, “water pollution”. This yielded sixty-four papers and excluded 639 papers.

EDCs were prioritised based on scoping the literature corpus: 27/64 (42%) of included environmental studies evaluated EDCs.

**Fig. 1:**
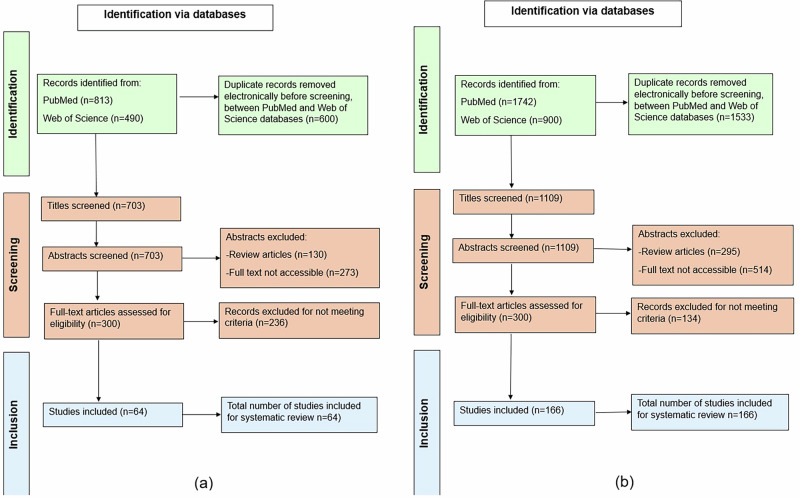
PRISMA flow chart used for the screening process of literature searches. **a** PRISMA flow chart used to select literature for the impact of the environment on endometriosis risk (**b**) PRISMA flow chart created for the screening process to select literature on genomic areas of interest in relation to endometriosis development.

A secondary literature search was conducted to identify genomic areas/ markers of interest involved in endometriosis. The keywords used in this search were “endometriosis” and “polymorphism”, “SNP”, “genetic polymorphism”, “variants”, “locus”, “GWA”, “Genome-wide”, “Genome wide”, “Genetic association study”. This yielded 166 papers and excluded 943 papers. Figure 1 shows the screening process of the literature searches as PRISMA flow charts.

From 57 candidates, five genes were pre-specified for a pilot, prioritising EDC responsiveness, pathway centrality, and expression at common implant sites. We focused on non-coding regulation (introns, untranslated regions, promoter-flanking, ±1 kb Transcription Start Site/Transcription End Site), given that EDCs more often perturb expression than protein sequence.

### 100,000 Genomics England database

The Genomics England (GE) 100,000 genome database [10, 11] was used as a part of an ongoing larger project “Genomic and chromosomal instability sequence markers in relation to fertility, early pregnancy, and cancers of the reproductive tissues” and included domains of Ovarian Cancer GE Research Network (GERN) and Endocrine and Metabolism GERN to obtain participants and analyse genomic data.

Participants from the GE Rare Disease Programs GRCh37 Participant Explorer were chosen by searching for a clinical diagnosis of endometriosis. All individuals were unrelated. No formal ancestry inference was undertaken in this pilot.

Inclusion Criteria: female participants aged eighteen to forty-three years at the time of recruitment, diagnosis of endometriosis recorded in medical history, availability of WGS data, inclusion of participants with endometriosis-related infertility and/or ovarian chocolate cysts.

Exclusion Criteria: individuals not assigned female sex at birth, participants over 43 years (to minimise cofounding by menopause-related genetic changes), presence of additional ovarian pathology, chromosomal abnormalities, haematological disorders or other reproductive tract malignancies, diagnosis of diabetes, immunological disorders, or hormonal conditions that could confound genetic associations, body mass index outside the range of 18.5–30 kg/m2 to minimize metabolic confounders' impact. The final study cohort included nineteen individuals meeting these criteria.

Each of the five selected genes were searched in GE per participant and single nucleotide variations (SNV), and insertion-deletion mutations (InDels) were collected.

This study focused on regulatory regions, introns, upstream, and downstream sequences, instead of coding regions, as environmental pollutants are more likely to affect gene expression than protein structure [12]. Targeting these regions enabled a more efficient and detailed investigation of relevant genomic variants.

### Variant selection and filtering

Within the five pre-selected genes (*IDO1*, *IL-6*, *CNR1*, *TACR3*, *KISS1R*), candidate variants were extracted in the GE IVA workspace using Ensembl [13] variant effect predictor consequence categories corresponding to regulatory sequence. Variants present in the endometriosis cohort were prioritised if they overlapped regulatory annotations and mapped to pathways implicated in EDC response. This yielded ten candidate non-coding variants for testing.

### Statistical analysis, linkage disequilibrium (LD) and Population branch statistic (PBS)

**Fig. 2:**
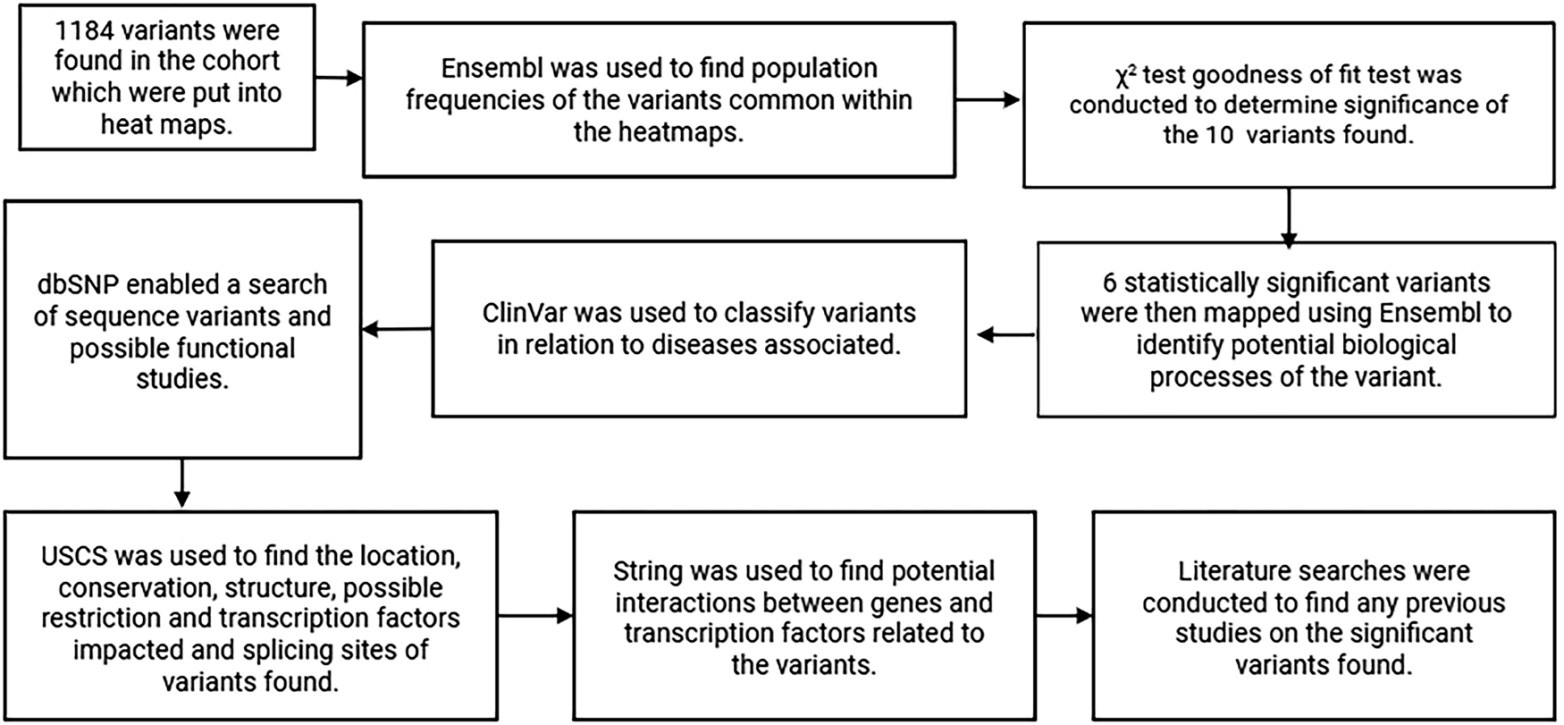
Workflow of methods followed for finding the impact and regulatory sequences of statistically significant variants.

Heatmaps were created using GraphPad Prism v10 [14] to visualise variants (heatmaps are available on request). Variant frequencies were compared between the general GE population, and the endometriosis cohort, using R v4.2.2 a χ² goodness of fit test for individual variants, with Fisher’s combined probability and small sample corrections relevant to data discovery. A Benjamini-Hochberg (BH) false discovery rate correction was applied to p-values, to account for multiple hypothesis testing controlling for false positives while maintaining statistical power (Fig. 2).

To confirm that variant enrichment was specific to the endometriosis cohort, nineteen randomly selected individuals without endometriosis from the GE database were screened using the same method. A Fisher's combined probability test compared variant frequencies across the endometriosis cohort, random group, and total GE population. Statistically significant variants were assessed for co-localisation to determine non-random clustering within the endometriosis cohort. LD analysis was conducted to assess the correlation between regulatory variants associated with endometriosis. For each pair of variants, we estimated the null probability of co-occurrence as the product of GE Total Population carrier proportions. We then compared the observed number of double-carriers using a one-sided tail test. Pairwise LD values (D’ and r²) were calculated for rs34880821 and rs2069840 in *IL-6* and rs806372 in *CNR1*, using data from the 1000 Genomes Project across multiple populations. LDpair and LDpop from LDlink [15–17] were used to determine linkage strength, with comparisons across African, East Asian, European, South Asian, and Admixed American populations. Results were analysed to evaluate population-specific evolutionary pressures and potential functional implications for immune regulation and pain sensitivity. We verified population allele frequencies for each rsID using Ensembl’s Population Genetics (1000 Genomes Phase 3) [13] and gnomAD; for per-population AF alongside LD context we used LDlink. Within the GE Research Environment, we also extracted GE Total allele counts (allele count/allele number) via IVA to compute allele frequency used in our χ² and BH procedures. For rare variants like rs76129761, super-population minor allele frequency is very low, making r² estimates likely imprecise; accordingly, we did not present population LD for that SNP and focused *CNR1* LD reporting on rs806372, which has stable super-population metrics.

To contextualise population differentiation of candidate variants, we computed PBS for 1000 Genomes super-populations (AFR (African population), EUR (European population), EAS (east Asian population)) using super-population allele frequencies from LDlink/1000G summaries. Pairwise F between populations was estimated from allele frequencies, transformed to branch lengths and combined. Negative PBS values were truncated to 0 for interpretation. PBS was calculated for rs76129761 and rs806372 (*CNR1*) and rs2069840 and rs34880821 (*IL-6*). Variants lacking reliable 1000G super-population coverage were not analysed.

### Regulatory sequence analysis

To identify statistically significant variant effects a search of the variants rsID was undertaken using ClinVar, dbSNP, ensemble, UCSC, and String [13, 18–26]. An extensive analysis was conducted using UCSC [23] and previous literature on other hominoids and the conservation of the statistically significant variants. All results are reported with adjusted *p*-values, and statistically significant associations are discussed in the context of biological plausibility and environmental interactions.

## Results

### Genomic findings of five selected genes and significant variant findings

Through using the literature search and consulting the criteria, the five genes chosen to be investigated further were *IDO1, IL-6*, *CNR1*, *TACR3*, and *KISS1R*.

Ten genetic variants were identified as potential contributors to endometriosis when compared to both the study population and the GE general population. Of these, 40% had a frequency of 0% in the Ensembl general population [13] but were present in the GE general population. This is potentially due to GE participants having a higher percentage of genetic variants through recruiting participants with known rare genetic conditions or specific diseases.

After conducting an χ² goodness of fit test for each of the ten variants found using the study cohort population frequency and GE total population frequency, variants, rs806372, rs76129761, rs2069840, rs34880821, rs933717388, and rs72643906 were found to be statistically significant when using a p value of 0.001. Following this a BH test was conducted, showing the variants are significant in the endometriosis cohort.

In the endometriosis cohort, 8/19 (47.4%) individuals carried both *IL-6* variants (rs2069840 and rs34880821). Using GE carrier proportions for these SNPs (6.24/19 and 4.80/19), the expected co-occurrence probability under independence 0.083, corresponding to 1.58 expected double-carriers in a nineteen-person sample. The observed count is therefore highly significant (*p* = 7.4 × 10⁻⁵). In the random nineteen-person group, 6/19 (31.6%) carried both, which is also higher than expected, though less pronounced (*p* = 3.4 × 10⁻³). This shows the co-localisation of rs2069840 and rs34880821 occurs much more frequently than would be expected by chance in both groups. Furthermore, both variants individually and together show significant enrichment in the endometriosis group. The elevated within-individual co-occurrence in random controls likely reflects underlying haplotype structure. Independence based expectations can underestimate co-occurrence when variants are correlated. We therefore treat co-occurrence qualitatively and emphasise case–control frequency contrasts for disease specificity. This suggests a potential biological interaction between these variants that may be particularly relevant to endometriosis.

When comparing the six significant variants in the endometriosis cohort compared to the random sample cohort and total GE populations, each of the variants is shown to be significantly higher in the endometriosis group, except rs76129761, which is shown to be higher in the random sample cohort. For rs76129761 (*CNR1*), counts were identical in cases and random controls (3/19 vs. 3/19). Both cohorts were nominally enriched versus the GE total population (χ² p = 0.023 in each), after correction only the endometriosis cohort remained significant (BH *p* = 0.046 vs. 0.092 in the random cohort). We therefore treat rs76129761 as a context-dependent candidate rather than protective, and we avoid interpreting random-cohort carriers as evidence against association. From the Fisher’s combined probability test between the endometriosis cohort and the random sample compared to the GE total population a combined χ², statistic; 44.37, with a p-value of 0.000013 (p < 0.001), showed strong evidence in overall frequency difference between the endometriosis cohort and the GE total population across all endometriosis variants. This suggests that the genetic profile of the endometriosis cohort significantly deviates from the general population, potentially indicating a unique genetic signature associated with endometriosis. Furthermore, the combined χ² statistic for the random sample cohort, 12.23, with a *p*-value of 0.427, showed no significant difference between the random sample cohort and the GE total population across all endometriosis variants. This lack of significant deviation suggests that the random sample’s variant frequencies are representative of the GE total population, serving as a control group.

Table 1 shows the variant profiles for the significant variants found within the endometriosis cohort.

**Table 1:** Variant profiles for the significant variants found within this cohort (created using UCSC [23]).

Variant	Associated gene.	Variant type/consequence.	Chromosome location.	Potential transcription factor (TF) binding site affected (if applicable).	Number of publications.	Other significant features.	Pollutant interference.	P-value from χ^2^ goodness of fit	BH-corrected p-value
rs72643906	*IDO1*	A > G Downstream variant	Chr8:39779157–399779157	*ZIC2* (Zic family member 2)	None	Highly conserved in mammals and vertebrates	BPA and TBBPA (Tetrabromobisphenol A) can decrease methylation of *IDO1*’s mRNA and DNA [31], and TCDD can increase IDO1 expression levels [49]. Exposure to BPA has been shown to decrease *ZIC2* expression [30].	0.00175665990877229	0.006
rs933717388	*KISS1R*	C > G Intron variant	Chr19:912365–912365	*ZNF707 (*Zinc finger protein 707*)* and *ZBTB11 (*Zinc finger and BTB domain-containing protein 11*)*	None	Conserved in humans	DEHP exposure influences regulators of HPGA by regulating GnRH (Gonadotropin hormone-releasing hormone) release altering *KISS1R* expression [42].	0.017530950708949900	0.036
rs34880821	*IL-6*	G > A Intergenic	Chr7:22775450–22775450	None	3	Conserved in humans Methylation site. Neandertal variant.	Exposure to TCDD can increase the expression of *IL-6* [49].	0.006042586245540780	0.024
rs2069840	*IL-6*	C > G Intronic	Chr7:22768572–22768572	None	12	Conserved in rhesus, mouse, and dogs.	Exposure to TCDD can increase the expression of *IL-6* [49].	0.020059606754631400	0.040
rs76129761	*CNR1*	CTCT > CT Intron	Chr6:88860156–88860156	*ZNF701 (*Zinc finger protein 701*), SOX4 (SRY-box transcription factor 4), SOX6 (SRY-box transcription factor 6), FOXD3 (*Forkhead box D3*)* and *SOX11 (SRY-box transcription factor 11)*.	None	Conserved in human, rhesus, mouse, and dogs.	Exposure to DiNP and BPA significantly increased the expression of *CNR1* [34]. Exposure to BPA can upregulate *SOX4* expression [38], *SOX6* and *SOX11* [37]. Exposure to BPA has been shown to decrease *FOXD3* levels [39].	0.023331593081759400	0.046
rs806372	*CNR1*	C > G Intron	Chr6:88846563–88866563	*SOX12 (SRY-box transcription factor 12)*.	3	Conserved in humans and rhesus. Splicing site; SIB locus ID: NC-000006-1470. 3 alternative transcripts within intron 1 (6q15) of *CNR1*. Denisovan variant	Exposure to DiNP and BPA significantly increased the expression of *CNR1* [34]. DEHP can increase *SOX12* expression levels [35].	0.003312817523356740	0.018

### LD analysis

**Fig. 3:**
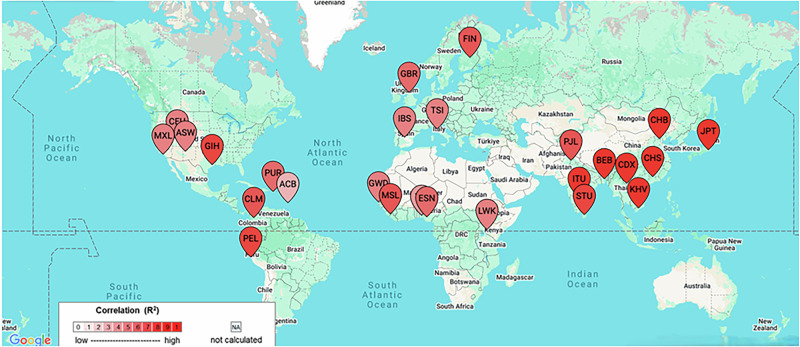
LD analysis of variants rs34880821 and rs2069840.

LD analysis revealed a linkage between Neandertal-derived *IL-6* variants (rs34880821 and rs2069840), in East Asians (D’ = 1.0, r2 = 0.9662), suggesting selective retention and potential immune regulation effects (depicted in Fig. 3), Europeans showed LD (D’ = 0.9234, r2 = 0.5794). The *IL-6* variant rs34880821, located at a Neandertal-derived methylation site, exhibits LD in East Asians (r² = 0.9662, D’ = 1.0), while showing LD in Europeans (r² = 0.5794) and South Asians (r² = 0.8104) [27]. In African populations, *IL-6* (rs2069840–rs34880821) shows D’ ≈ 0.875, r²≈0.482, while *CNR1* (rs76129761–rs806372) is near-zero, likely due to the absence of Neandertal introgression.

The Denisovan-influenced *CNR1* variant (rs806372) exhibited LD in East Asians (D’ = 0.8004) but LD in Europeans (D’ = 0.4607) and South Asians (D’ = 0.1459), suggesting population-specific evolutionary pressures. African populations had negligible LD for both variant pairs, supporting the hypothesis that these associations arose post-migration due to archaic human introgression.

These findings highlight six regulatory variants significantly enriched in the endometriosis cohort. Notably, the *IL-6* variants rs2069840 and rs34880821 not only co-occurred at a rate well above chance but also exhibit population-specific LD consistent with archaic hominin ancestry and potential immunoregulatory roles. Other significant variants in *CNR1*, *IDO1*, and *KISS1R* were associated with transcription factor binding sites and are in genomic regions shown to be responsive to EDCs.

Consistent with LD patterns, *CNR1* rs806372 showed a strong EAS-specific PBS (~0.49), with much smaller values in AFR and EUR. See supplementary material for more detail.

## Discussion

Many sufferers of endometriosis are subject to misdiagnosis and delays due to limited understanding of early-stage risk factors. To obtain a better understanding of genetic factors predisposing to endometriosis development, two literature searches and interrogation of the GE 100,000 genome database were conducted.

Using a highly targeted multilevel approach, five genes have been characterised as potential targets in the developing endometriosis pathway, containing variants or having altered expression levels, with six variants found as statistically significant in our cohort population when compared to GE. This builds on the work conducted by Sapkota, Rahmioglu, and Zondervan [7–9], who collectively found forty-two SNPs linked to endometriosis.

### IDO1

The downstream variant of *IDO1* rs72643906 is found to be rare in the general population, and is significantly higher in the study cohort, indicating the variant may influence endometriosis development through altering *IDO1* expression levels. Specifically, Brooks [28] found that immune system activation increases *IDO1* expression. Furthermore, the UCSC [23] database showed an association with an elevated risk of COVID-19, and therefore, is potentially disadvantageous to the immune system, which is seen to be associated with endometriosis patients [4]. The UCSC database [23] showed rs72643906 to alter the motif of the transcription factor ZIC2, which regulates tissue expression, this potentially modifies the ZIC2 motif to prevent the transcription factor from binding, therefore hypothetically increasing the expression levels of *IDO1* and ZIC2. Furthermore, elevated *IDO1* expression has been found in endometriosis patients [29], showing a potential link between rs72643906 and endometriosis. However, when Bisphenol A (BPA) is introduced through the environment, *IDO1* and ZIC2 expression levels are decreased [30, 31] potentially creating a new dysregulated baseline effect of rs72643906. EDC exposures are considered baseline shifting rather than uniformly opposing variant effects: in carriers, exposures can establish a new dysregulated set-point, BPA lowering *IDO1*/ZIC2 despite a variant-predisposed higher baseline and related to possible predictions of exposure dependent penetrance with some additivity.

### CNR1

The *CNR1* variants rs76129761 and rs806372 were found to be significant within the study cohort; rs76129761 is deleterious, and rs806372 has a potential splicing site implicating three alternative transcripts within intron 1 of *CNR1*. Therefore, it is possible these variants potentially increase *CNR1* expression levels, leading to endometriosis development, with Allam [32] finding increased *CNR1* levels in endometriosis patients when compared to controls.

Variant rs76129761 was found to be high in the random control group as well as the endometriosis cohort. This may be due to undiagnosed subclinical conditions. Furthermore, the UCSC database [23] found this variant to be high within Northern Sweden, Estonian, and Korean populations and is highly conserved in humans, rhesus monkeys, mice, and dogs. This potentially indicates an advantageous effect of this variant which when exposed to environmental factors may become disadvantageous.

The UCSC database [23] shows rs806372 to be a Denisovan variant, which may be well established in populations where Denisovans and sapiens interact, being randomly selected until the onset of EDCs in our modern society [33]. The consequence of this may have been exposure to Di-(2-ethylhexyl) phthalate (DEHP) and BPA, which has been found to increase *CNR1* expression [34].

The UCSC database [23] also shows rs806372 to lie on the binding site of SOX12. This may alter the motif, disrupt its binding and lead to increased *CNR1* and decreased SOX12 expression levels. However, environmental DEHP exposure has been shown to elevate both SOX12 and *CNR1* levels, as supported by expression studies [35, 36].

Variant rs76129761 lies on the binding site of five transcription factors; ZNF701, SOX4, SOX6, FOXD3 and SOX11, which may cause increased expression levels of the these and *CNR1* gene. Furthermore, environmental BPA exposure increases SOX4, SOX6, and SOX11 [37, 38] and decreases FOXD3 expression levels [39].

These variants, when exposed to DEHP and BPA may establish dysregulation leading to endometriosis development. EDC exposures are considered baseline shifting rather than uniformly opposing variant effects: in carriers, exposures can establish a new dysregulated set-point DEHP elevating both SOX12 and *CNR1* despite a motif-disrupting allele and related to possible predictions of exposure dependent penetrance with some additivity.

### KISS1R

The *KISS1R* intronic variant rs933717388 is a potentially novel variant (no literature or records referencing this variant) and was found to be significantly higher in the study population. The UCSC database [23] found rs933717388 to lie on the binding sites of ZNF707 and ZTB11. The variant rs933717388 may bind to the sites of transcription factors ZNF707 and ZTB11 altering their motifs and potentially hindering their binding. Since ZBTB11 is a silencing transcription factor, reduced binding could increase *KISS1R* expression. This upregulation may disrupt reproductive function and promote endometriotic cell metastasis [40], Blasco [41] supports this, reporting elevated *KISS1R* levels in granulosa cells of endometriosis patients. Both *KISS1R* and transcription factor levels are potentially increased in the presence of rs933717388, and DEHP exposure further raises *KISS1R* expression [42], though its effect on ZNF707 and ZTB11 remains unstudied.

### IL-6

Intronic variants rs2069850 and rs34880821 of the *IL-6* gene were found to be significant within the study cohort. These variants have been found to colocalise in the endometriosis cohort, which suggests a potential biological interaction between these variants that are potentially relevant to endometriosis. UCSC [23] showed rs34880821 to be a Neandertal methylation site. Therefore, the reference allele was methylated in Neandertals and Denisovans, but this variant abolishes the methylation site, so rs34880821 may exacerbate endometriosis development due to the disease being associated with dysregulation of the immune and inflammatory system by aberrant silencing of the area and continuous expression. This probability of heightened *IL-6* expression in endometriosis patients is further validated in research by Li [43], finding an increase in *IL-6* expression levels when compared to controls.

### The intersection of ancient genetic variants, epigenetic regulation, and modern environmental pollutants in endometriosis susceptibility

Our findings highlight how ancient genetic variants inherited from Neandertals and Denisovans, epigenetic regulation, and modern industrial pollutants may potentially converge to shape population-specific risks for endometriosis. The LD analysis of *IL-6* and *CNR1* regulatory variants suggests that evolutionary pressures involving immune regulation and inflammatory responses, when combined with modern environmental exposures, may amplify disease susceptibility [27, 44].

Neandertal introgression has significantly influenced *IL-6* regulation, particularly in East Asian populations. This suggests that Neandertal introgression may have played a role in shaping *IL-6* regulatory pathways, particularly in East Asians, where it remains strongly linked. Given that EAS also have the highest reported prevalence of endometriosis (~15.4%), it is likely that these genetic variants contribute to heightened inflammatory responses, immune dysregulation, and fibrotic lesion formation [45].

The functionality for the Neandertal-derived methylation at rs34880821 may indicate another subtle contributor to endometriosis risk. Methylation usually functions as a gene-silencing mechanism, regulating cytokine levels to prevent excessive inflammation [46]. If methylation is lost, *IL-6* expression could become hyperactive, leading to chronic immune activation, sustaining peritoneal inflammation, fibrosis, and deep-infiltrating endometriosis, as *IL-6* is known to drive fibrotic remodelling in reproductive tissues. It also may account for heightened neuroinflammation, potentially explaining increased pain sensitivity in East Asian endometriosis patients [45].

Similarly, the Denisovan-derived rs806372 variant in *CNR1*, which is involved in immune modulation and pain perception, exhibits LD in East Asians [26]. Since *CNR1* plays a key role in pain signalling and inflammatory responses, this Denisovan-derived variant may enhance pain sensitivity in individuals with endometriosis, particularly in EAS [47].

While these ancestral variants may have once provided immune advantages in prehistoric environments, modern environmental factors may be reversing these evolutionary benefits, transforming once-adaptive immune responses into drivers of chronic disease [45].

Exposure to EDCs, such as BPA, phthalates, and dioxins, has been shown to demethylate immune regulatory genes, including *IL-6*, leading to excessive cytokine production [48]. If Neandertal-derived *IL-6* regulatory variants are already prone to overactivation, additional EDC-induced demethylation could further escalate inflammatory signalling, worsening lesion development. This suggests a gene-environment interaction, where modern industrial chemicals exacerbate genetic predispositions inherited from archaic human ancestors.

In contrast to EAS and EUR, AFR exhibit significantly lower LD for these *IL-6* and *CNR1* variants (D’ < 0.02, r² < 0.001), suggesting these genetic associations arose post-migration due to Neandertal and Denisovan introgression [27]. There is limited available data on the prevalence of endometriosis in AFR, and estimates may vary due to underdiagnosis.

PBS profiles indicate that the *IL-6* regulatory pair is most differentiated in EUR and the *CNR1* signal is strongest in EAS, consistent with the directionality of the LDpop-derived allele frequency differences. Although PBS alone does not prove selection there is concordance with functional hypotheses that warrants further study.

This study supports a novel perspective for endometriosis susceptibility, in which ancestral genetic variants interact with modern environmental pollutants to modulate immune regulation, chronic inflammation, and pain perception across populations.

### Limitations and future directions

While this study provides novel insights into the genetic and environmental factors influencing endometriosis susceptibility, several limitations must be acknowledged. We reference archaic ancestry as context (haplotypic/methylation signatures) rather than proof of inheritance. Future work will include outgroup allele-state checks and selection statistics to test evolutionary hypotheses directly. The sample size was limited, particularly in younger individuals under twenty-nine years old. Additionally, family-based cascade genetic testing was not included, preventing the evaluation of heritability and potential familial aggregation of risk variants. Another limitation is the reliance on medical records to infer endometriosis staging, which introduces the potential for misclassification bias. Furthermore, while this study identified significant associations between regulatory variants and endometriosis risk, functional validation through in vitro and vivo models is required to confirm their biological relevance.

Further research should focus on expanding the study cohort to include a larger and more diverse population to improve the statistical power of genetic associations and assess whether these findings are consistent across different ethnic backgrounds. Family-based studies incorporating cascade genetic testing could help clarify inheritance patterns and the potential contribution of additional rare variants to endometriosis risk. Given the discovery phase design, findings are hypothesis-generating. Replication in large, ancestry-stratified cohorts (e.g., UK Biobank/other databases) will be pursued in a subsequent protocol and is beyond the scope of this pilot. Further functional studies are necessary to evaluate how the identified regulatory variants influence gene expression and immune signalling pathways, particularly in response to EDCs. Integrating environmental exposure data with genomic analysis would provide a more comprehensive understanding of how genetic and environmental factors interact in the development and progression of endometriosis. Potentially enabling computational modelling of gene regulatory networks under varying EDC exposures to predict combined outcomes and guide specific functional validation experiments. These future directions will be critical for translating genetic discoveries into practical applications for early diagnosis and personalised treatment strategies.

## Conclusion

This study provides a novel perspective on the genetic and environmental interplay driving endometriosis susceptibility, highlighting the influence of ancient regulatory variants and modern industrial pollutants. This research identified statistically significant regulatory variants in *IL-6*, *CNR1*, *IDO1*, and *KISS1R* that may contribute to endometriosis risk through interactions with EDCs. These findings aim to bridge the gap between genetic predisposition, and environmental exposures, shedding light on how Neandertal and Denisovan introgressed variants in immune and pain-regulatory genes may influence disease susceptibility in modern populations.

This is the first study to systematically explore how ancient genetic signatures, epigenetic regulation, and contemporary environmental pollutants intersect in the pathophysiology of endometriosis. These results lay the first steps for future precision medicine approaches, where genetic screening combined with environmental risk assessment may improve early detection and personalised intervention strategies. Moving forward, functional validation of these regulatory variants and their interaction with endocrine disruptors will be crucial in understanding their mechanistic role in endometriosis onset and progression. These findings enhance our understanding of endometriosis as a multifactorial disease but also provide a framework for future research integrating evolutionary genetics, environmental health, and reproductive medicine.

## Supplementary information

Supplementary data PBS

Supplementary data (LD)

## Data Availability

Research on the de-identified patient data used in this publication can be carried out in the Genomics England Research Environment subject to a collaborative agreement that adheres to patient led governance. All interested readers will be able to access the data in the same manner that the authors accessed the data. For more information about accessing the data, interested readers may contact research-network@genomicsengland.co.uk or access the relevant information on the Genomics England website: https://www.genomicsengland.co.uk/research.
