# Supplementary material for: Endometriosis - on the intersection of modern environmental pollutants and ancient genetic regulatory variants
Source: Eur J Hum Genet. 2025 Nov 20;34(2):243–51. doi: 10.1038/s41431-025-01977-9 (PMC12858970; doi:10.1038/s41431-025-01977-9)
Supplement: Supplementary file 1 — Supplementary data PBS [file 41431_2025_1977_MOESM1_ESM.docx]

Supplementary Methods: Population Branch Statistic (PBS)

To contextualise population differentiation of candidate variants, we computed PBS for 1000 Genomes super‑populations (AFR, EUR, EAS) using super‑population allele frequencies from LDlink/1000G summaries. Pairwise FST between populations was estimated from allele frequencies, transformed to branch lengths and combined. Negative PBS values were truncated to 0 for interpretation. PBS was calculated for rs76129761 and rs806372 (CNR1) and rs2069840 and rs34880821 (*IL-6*). Variants lacking reliable 1000G super‑population coverage were not analysed. These FST estimates were transformed into additive distances using a log10 transform (−log10(1 − FST)), and branch lengths were combined to yield PBS for each focal population X: PBS_X = (T_XY + T_XZ − T_YZ)/2. Variant‑specific allele frequencies (AF) were obtained from 1000 Genomes Phase 3 super‑populations (AFR, EUR, EAS). Variants rs72643906 (*IDO1*) and rs933717388 (*KISS1R*) lacked reliable AFs across these populations and were not included.

Table S1 – 1000 Genomes super‑population allele frequencies and PBS values. Frequencies are alternate‑allele frequencies

| **Variant** | **Alt allele** | **AFR AF** | **EUR AF** | **EAS AF** | **PBS AFR** | **PBS EUR** | **PBS EAS** | **Gene** |
| --- | --- | --- | --- | --- | --- | --- | --- | --- |
| *CNR1* rs76129761 (allele C) | C | 0.0159 | 0.0398 | 0.0099 | -0.0033 | 0.013905 | 0.004714 | CNR1 |
| *CNR1* rs806372 (allele C) | C | 0.0765 | 0.1193 | 0.501 | 0.085855 | -0.07543 | 0.491669 | CNR1 |
| *IL-6* rs2069840 (allele A) | A | 0.1 | 0.2525 | 0.0605 | -0.02814 | 0.111623 | 0.038768 | IL6 |
| *IL-6* rs34880821 (allele G) | G | 0.15 | 0.332 | 0.0625 | -0.06223 | 0.157137 | 0.103378 | IL6 |

rs806372 shows strong East‑Asian specific differentiation, while rs2069840 and rs34880821 show moderate European‑specific differentiation. rs806372 has a high PBS in East Asians (~0.49), consistent with strong differentiation in the EAS lineage relative to Africans and Europeans. Conversely, rs2069840 and rs34880821 show moderate PBS in Europeans (~0.11 – 0.16) and smaller PBS in East Asians, suggesting a more pronounced European‑specific shift for these IL6 variants. Variants with negative PBS values in a population (e.g., rs806372 in EUR) indicate that the focal branch length is shorter than expected.

*Sources - Yi X, et al. Sequencing of 50 Human Exomes Reveals Adaptation to High Altitude. Science. 2010;329:75–78.*

*Shpak M, et al. The Precision and Power of Population Branch Statistics in Detecting Local Adaptation. Genome Biology and Evolution. 2024.*
